# The role of Gabapentin oral solution in decreasing desflurane associated emergence agitation and delirium in children after stabismus surgery, a prospective randomized double-blind study

**DOI:** 10.1186/s12871-018-0533-5

**Published:** 2018-06-20

**Authors:** Ahmed A. Badawy, Samaa A. Kasem, Doaa Rashwan, Tarek Al Menesy, Ghada Adel, Ali M. Mokhtar, Yasmin A. Badawy

**Affiliations:** 1 Anesthesia department, Faculty of medicine, Cairo University, Giza, Egypt; 2 Anesthesia department, Faculty of medicine, Beni Suef University, Beni Suef, Egypt; 3 Faculty of Medicine, Cairo University, Giza, Egypt

**Keywords:** Gabapentin, Desflurane, Emergence agitation, Strabismus

## Abstract

**Background:**

Short acting inhalational anesthetic (Desflurane) produces emergence agitation (EA) in pediatrics with an incidence up to 80%. The aim of the present study was to examine the role of Gabapentin oral solution in attenuating desflurane associated EA in children after strabismus surgery under general anesthesia.

**Methods:**

Seventy patients, 2–6 years old, scheduled for strabismus surgery were randomly allocated into two groups (35 each); Control group (c): received 5 ml of oral strawberry juice (placebo) and Gabapentin group (G) received 5 mg/Kg gabapentin oral solution in 5 ml strawberry juice, 1 h before anesthesia. Patient separation, cooperation, emergence incidence and emergence severity were assessed. Also time to extubation and time to emergence, duration of PACU stay, PONV and number of patients required meperidine postoperatively were recorded.

**Results:**

Duration to extubation and duration to emergence were statistically prolonged in gabapentin group compared to the control group. The incidence of EA and its severity were reduced in gabapentin group with more tendencies to be asleep and less attentive. More patients in the control group required postoperative meperidine to reduce crying and agitation.

**Conclusion:**

Oral gabapentin 5 mg/kg reduced the incidence and severity scoring of emergence agitation (by 20%) with more tendencies for sleeping with preserved response to stimuli in PACU.

**Trial registration:**

Number: NCT03347916, date: November 17, 2017, retrospectively.

## Background

Emergence agitation (EA) and emergence delirium (ED) in pediatrics during recovery from general anesthesia has been defined as a state of dissociated consciousness with excitement, irritability, uncooperation, thrashing, crying, moaning or incoherence [1, 2].

Inhalational anesthetics when used in pediatric patients undergoing surgery under general anesthesia can often produce emergence agitation (EA) and emergence delirium (ED) with the incidence ranges from 18 to 80% [3].

The risk factors for post-anesthesia EA and ED in pediatrics include; pre-school age, previous anesthesia, type of surgery (ophthalmology and otorhinolaryngology) and rapid recovery from inhalational anesthetics with faster recovery profile [4].

Desflurane, due to its low solubility in blood, shows a unique characteristic of rapid wash out and faster recovery among the modern inhalational anesthetics, which is associated with a comparable incidence of (EA) compared with sevoflurane and isoflurane, specially in children [5, 6].

Gabapentin, a gamma-aminobutyric acid analog, binds the voltage-gated calcium channels of the dorsal root ganglion, at α2-δ subunit [7]. Gabapentin binding to these channels inhibits the release of excitatory neurotransmitters preventing the propagation of painful stimuli which makes its use attractive in treatment of postoperative pain and agitation with less side effects compared with opioids and benzodiazepines [8].

Gabapentin has been used in controlling acute perioperative conditions like preoperative anxiety, intraoperative attenuation of hemodynamic response to noxious stimuli and postoperative pain, delirium and nausea and vomiting [9].

Strabismus is the most common ophthalmic surgical procedure in pediatric patients with postoperative little to moderate pain but high incidence of emergence delirium and agitation (40–86%) [10–13].

Recent studies [8, 14], (*one of them carried out in NICU at Cleveland Clinic Children’s Hospital in Cleveland, Ohio)*, examined the use of gabapentin for pain and agitation in Neonates and Infants in a Neonatal ICU encouraged us to use gabapentin in children.

To our knowledge there were no reports evaluating the effect of gabapentin on emergence agitation after desflurane, therefore ***the aim of this study*** was to evaluate the effect of preoperative oral gabapentin 5 mg/kg given 1 h before surgery on emergence agitation after desflurane anesthesia in children after strabismus surgery.

## Methods

After obtaining the ethical committee approval of Kuwait Specialized Eye Center and an informed consent from the parents, Seventy pediatric patients (2–6 years old), with an *American society of Anesthegiologists* physical status (ASA) I-II who were undergoing strabismus surgery *(for more than one muscle)* were enrolled in this randomized double –blind study from January to November 2017. The present study was taken place in ***Kuwait Specialized Eye Center*** *(where all patients recruited)* and was registered at *ClinicalTrials.gov* with Identification number**:** ***NCT03347916*** and registration date*:* November 17, 2017, retrospectively.

*Exclusion criteria* were failure to obtain consent, mental retardation or developmental delay, epilepsy, psychiatric or neurological diseases that impair communication, current use of gabapentin, psychotropic or opioids and history of premature birth.

Patients were randomly (using sealed opaque numbered envelopes indicating the group of each patient, carried out by an independent anesthesiologist) allocated into one of two groups (35 patients each):

- **Control group (C)** [*n = 35*]: received strawberry juice 5 ml volume, one hour before induction.
- **Gabapentin group (G)** [*n = 35*]: received gabapentin (*Neurontin oral solution 250 mg/ml, Pfizer, USA*) **5 mg/kg** [8, 14] mixed with strawberry juice to constitute 5 ml volume, one hour before induction.

Anesthesia nurse assigned to receive the patient in the operating room was trained to use and apply the ***“separation score*”** [15] before separating the child from his parent, [**1 = Excellent**: *happily separated*, **2 = Good**: *separated without crying*, **3 = Fair**: *separated with cryin*g, **4 = Poor:** *need for restraint*]. If the separation was unsuccessful *(score 3 and 4)*, either of the parents was allowed to attend the induction and also to stay with the patient *(postoperatively)* in the PACU till discharge.

Upon arrival to operating room, standard monitors were attached; (5 leads ECG, pulse oximetry, suitable sized non-invasive blood pressure cuff, capnography and surface temperature probe). Induction was carried out using sevoflurane 8 vol% in oxygen 100% via face mask till loss of consciousness when intravenous cannula (24–22 gauge) was inserted and intravenous (IV) Dextrose 5% in normal saline 0.9% started. During induction, the patient cooperation was observed and scored according to Pandit et al. ***“cooperation score”*** [12]: [***1 =*** *Cooperative,* ***2 =*** *mildly resistant,* ***3 =*** *Resistant to placement of face mask*]. Bispectral index *(BIS VISTATM, Aspect Medical System Inc., MA, USA)* monitoring pediatric strip was attached to the forehead and inhalational anesthetic switched to desflurane, titrated to maintain BIS score 40–60 with hemodynamics maintained within 20% of the pre-induction values. When anaesthesia was deep enough, a laryngeal mask air way was inserted and supportive mechanical ventilation was initiated *(Pressure support ventilation)* with relatively high respiratory rate, when needed *(an investigator personal experience)* to reduce the need for neuromuscular blockade and to prevent airway peak pressure from exceeding 18–20 cmH_2_O, while keeping the end-tidal carbon dioxide at 34–36 mmHg. After induction, all patients were given dexamethasone 0.15 mg/kg *(as prophylaxis for PONV)* and Diclofenac 12.5 mg suppository. Before cleaning the eye, tetracaine 0.5% jelly *(TetraVisc, Altaire Pharmaceuticals, Inc. NY., USA)* was applied to the conjunctiva, then before starting surgery the surgeon was instructed to inject sub-conjunctival 1–2 ml of bupivacaine 0.5% which was repeated after closure of conjunctiva *(at the end of surgery)* to assure pre-emptive and preventive analgesia. Lastly, antibiotic eye drops were applied without an eye patch to exclude agitation due to closed eye. Later, antibiotic ointment was applied just before discharge from the PACU. After closure of the conjunctiva, desflurane was discontinued and pressure support was switched to spontaneous breathing with manual assistance and laryngeal mask was removed after cough, gag reflex, grimace, or purposeful movement. The patient was given oxygen 100% via face mask before shifting to post-anesthesia care unit (PACU), where the patient was monitored and observed till fulfilling *Aldrete* scoring 9 or more [16], when discharged to the ward.

In the PACU, emergence condition was scored using ***“emergence agitation scale”*** [12]: [***1 =*** *Obtunded with no response to stimuli*; ***2 =*** *Asleep, but responsive to movement and stimuli;* ***3 =*** *Awake and appropriately responsive*; and ***4 =*** *Crying and difficult to console*; ***5 =*** *Wild thrashing behaviour that requires restraint*]. A score 4 or 5 was treated by IV meperidine 0.5–1 mg/kg increments. Also postoperative nausea *(PONV)* and vomiting was observed and scored using 4-degree scale [17]: [***0 =*** *absence of nausea and vomiting;* ***1 =*** *nausea only;* ***2 =*** *single emetic episode;* ***3 =*** *multiple emetic episodes*]. Any degree of (PONV) was treated using ondansetron 0.15 mg/kg.

Patients were discharged from PACU, if they have EA score 3 or less after 30 min from the last meperidine dose. Any complications were observed, documented and managed appropriately.

The following were observed *(by anaesthetist blinded to the studied drugs)*:

- Patient separation, cooperation and emergence agitation incidence and scores *(as primary outcomes)*.
- Demographic data *(age, sex and body weight)*, duration of surgery *(the time between application and removal of the eye speculum)*, duration of extubation *(the time from discontinuation of the anesthetic to removal of LMA),* duration of emergence *(The time from the discontinuation of anesthesia till the time of spontaneous eye opening or to verbal command)*, duration of PACU stay *(the time from arrival to PACU to discharge to ward),* number of patients required post operative meperidine and postoperative nausea and vomiting *(PONV)*.

### Statistical analysis

Sample size calculation was done after a pilot study where the difference in incidence of EA between groups was 30%. By using the power analysis 80% at level of significance 0.05 [α = 0.05 and β = 0.8] and a drop-out rate 10%, the number of patients in each group was 34.

**Fig. 1:**
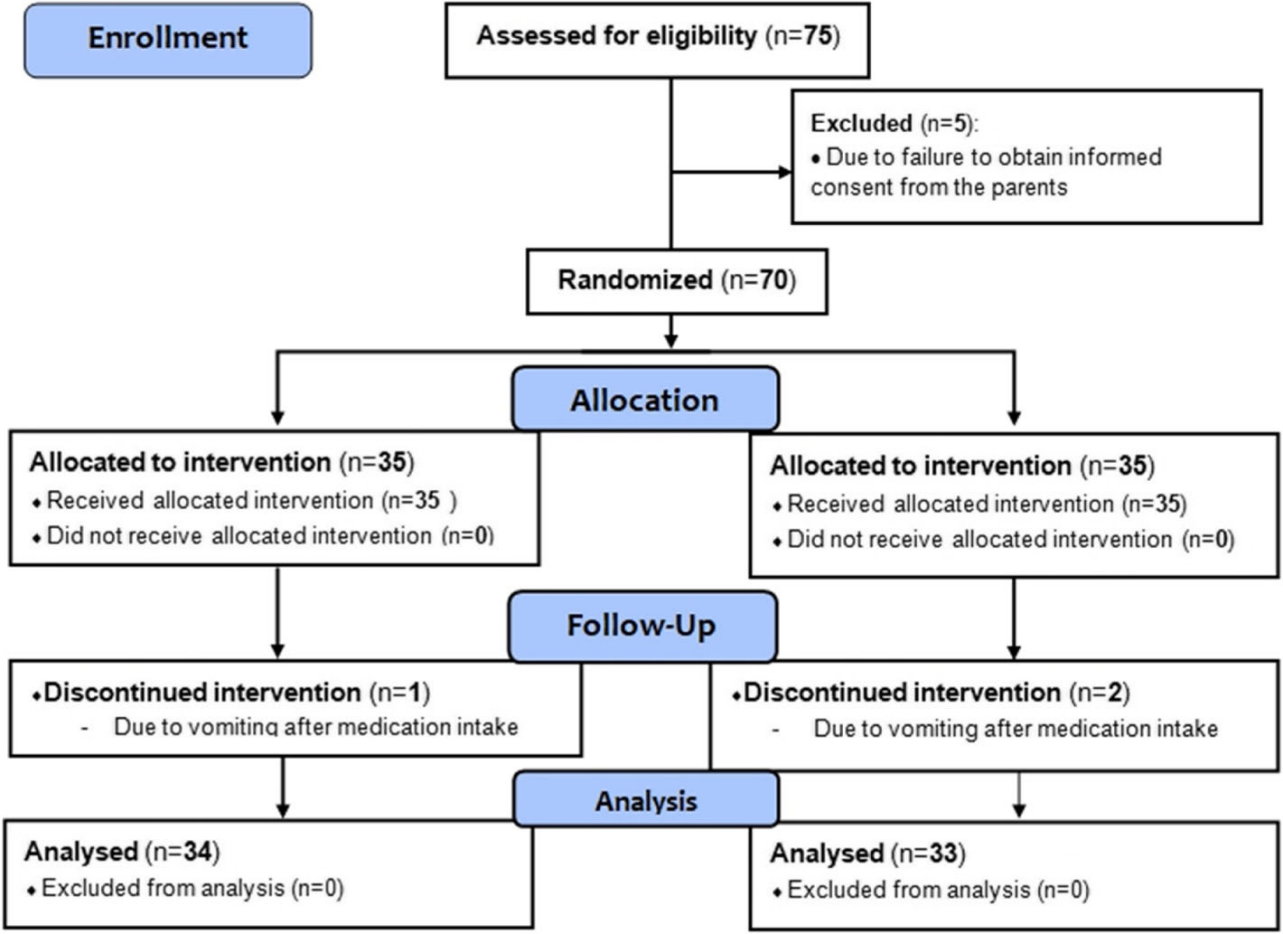
Flow diagram of the study

Statistical analysis was performed using PASW Statistics 18 (SPSS Inc., Chicago, IL, USA). Data were reported as [mean ± SD], [median (range)] or number and percentage. Parametric data were analysed using an unpaired Student’s t-test. Ordinal data were analysed using the Mann-Whitney ranked sum test. Nominal data were analysed using either the chi-square or Fisher’s exact test (Fig. 1).

## Results

The results of the present study showed no statistically significant difference between the two studied group as regards the demographic data, the operative duration or duration of PACU stay. Duration to extubation was statistically significant prolonged in Gabapentin group compared to the control group [4.9 ± 0.6 and 4.4 ± 0.7 min] respectively. Also duration to emergence showed statistically significant prolongation in G group in comparison to C group [7.4 ± 07 and 6. 9 ± 0.4 min] respectively (Table 1).

**Table 1:** Demographic and peri-operative data of the studied groups. Data represented as [mean ± SD] or Number

	Group (C) *(n = 34)*	group (G) *(n = 33)*	*P value*
Age *(year)*	4.2 ± 1.2	3.7 ± 1.4	0.07
Sex *(Male/Female)*	26/7	23/9	0.8
Body weight *(kg)*	17 9 ± 3.3	16.8 ± 3.2	0.21
Duration of surgery *(minutes)*	41.8 ± 3. 9	43.1 ± 4.6	0.12
Duration of extubation *(minutes)*	4.4 ± 0.7	4.9 ± 0.6*	0.002
Duration of emergence *(minutes)*	6. 9 ± 0.4	7.4 ± 07*	0.000
Duration of PACU stay *(minutes)*	31.6 ± 3.2	32..8 ± 3.7	0.075

*Group (C) = control, group (G) = Gabapentin and PACU = post-anesthesia care unit*

*Statistically significant compared to group (C), [p ˂ 0.05]

Separation score (from parents) and cooperation score (to face mask placement for induction) showed no statistically significant difference between the two group, while emergence agitation score was significantly lower in G group compared to C group (*p ˂ 0.05),* (Table 2)*.*

**Table 2:** Separation, Cooperation and Emergence agitation (EA) scores. Data represented as [median (range)]

	Group (C) *(n = 34)*	group (G) *(n = 33)*	*P value*
Separation score	3 (1–4)	2.0 (1–4)	0.09
Cooperation score	2 (1–3)	2 (1–3)	0.1
Emergence Agitation score	4 (2–5)	3 (2–5)*	0.006

*Group (C) = control and group (G) = Gabapentin*

*Statistically significant compared to group (C), [p ˂ 0.05]

The overall incidence of EA was higher in the control group [18/34] compared to the gabapentin group [10/33]. The Grades of agitation showed that none of patients in both groups was *Obtunded with no response to stimuli,* grade (1) and only two patients in group (C) and one patient in group (G) showed maximum agitation *(thrashing behaviour that requires restraint),* grade (5). Patients in G group showed statistically significant higher tendency to be sleepy, grade (2) (*asleep, but responsive to movement and stimuli)*, while those in group (C) showed statistically significant tendency to grade (4), (*Crying and difficult to console),* (Table 3), and hence, more patients required meperidine to control in the PACU, (Table 4).

**Table 3:** Incidence and Grades of emergence agitation (EA). Data represented as [number (percentage %)]

	Group (C) *(n = 34)*	group (G) *(n = 33)*	*P value*
Overall incidence of agitation (Grade ≥ 4)	18 (52.9%)	10 (30.3%)*	0.03
Grades of agitation			
• Grade 1*(minimum)*	0 (0%)	0 (0%)	0.6
• Grade 2	6 (17.6%)	16 (48.4%)*	0.000
• Grade 3	10 (29.4%)	7 (21.2%)	0.2
• Grade 4	16 (47%)	9 (27.2%)*	0.03
• Grade 5 *(maximum)*	2(5.8%)	1 (3)	0.5

*Group (C) = control and group (G) = Gabapentin*

*Statistically significant compared to group (C), [p ˂ 0.05]

**Table 4:** Postoperative meperidine and incidence of PONV. Data represented as number (percentage %)

	Group (C) *(n = 34)*	group (G) *(n = 33)*	*P value*
Number of patients required postoperative meperidine *[No. (%)]*	18 (52.9%)	10 (30.3%)*	0.03
Incidence of PONV *[No. (%)]*	3 (10%)	2 (6.6%)	0.32

*Group (C) = control and group (G) = Gabapentin. PONV = postoperative nausea and vomiting*

*Statistically significant compared to group (C), [p ˂ 0.05]

The incidence of PONV showed no statistical difference between the two groups (Table 4). No other side effects or complications were recorded.

## Discussion

Modern inhalational anesthetics like sevoflurane and desflurane are characterized by rapid wash out and recovery profile, that is associated with high incidence of emergence agitation (EA) up to 80%, when used as sole anesthetic agents, specially in children [4]. The exact mechanism of EA after inhalational anesthesia is not clear. Some explanations for this phenomenon are the rapid wash out of anesthetics, lack of adaptation to the environment after waking up, pain sensation and separation from parents [18].

The present study showed that gabapentin statistically extended the duration to extubation and to emergence *(without clinical significance)* and reduced the overall incidence and severity score of emergence agitation (EA). As regards the grades of emergence agitation in PACU, gabapentin showed to increase the tendency for sleeping with preserved response to stimuli, compared to the control group. None of the patients in both groups showed obtundation and only three patients were maximally agitated.

To our knowledge, gabapentin was not studied as regards the small point, focused on in the present study, which is its use to attenuate the agitation during emergence from desflurane inhalational anesthetic. Lack of literature about the point of our interest, represented some difficulty in choosing the proper dose suitable for our pediatric population. Before starting our experiment, only one study published in 2016; Edwards L et a., [14] studied the gabapentin use to control hyperalgesia and agitation in neonatal intensive care unit *(NICU)* using an average dose of 10–15 mg/kg/day. Later, in June 2017; Gretchen L et al., [8] published their study in the same field and they mentioned that gabapentin dose used was 10–16 mg/kg/day. Due to our limited experience with gabapentin we chose a lower dose (5 mg/kg) given only once.

Amin SM and Amr YM [19] examined the use of gabapentin in pre-emptive pain control after adeno-tonsillectomy in children. Gabapentin showed superior pain control to paracetamol. Some differences exist between their work and the present study as they assessed pain (not agitation) using visual analogue scale which needs some cooperation from the patient to express himself. Also they used higher dose of gabapentin (10 mg/kg) depending only on the systemic analgesics without any local anesthetic infiltration. Their study should 50% reduction of postoperative total meperidine requirement in gabapentin with longer duration for the first request of analgesic.

A similar study to the present work done by Kim J. et al., [4] studied the effect of dexmedetomidine on the reduction of emergence agitation in children operated for strabismus surgery under desflurane anesthesia and showed that agitation score and maximum agitation in PACU were significantly reduced using intraoperative dexmedetomidine infusion (0.2 μg/kg/h) s in addition to fentanyl (1 μg/kg) compared to fentanyl only. They mentioned that, their patients could not open their eyes in PACU, because of pain and frequency of rescue fentanyl in the control group was high compared to the present study. This difference could be explained by the preemptive and preventive subconjunctival bupivacaine used in our patients.

Another work done by Kim KM et al., [20] who examined the effect of intravenous preoperative midazolam [0.1 mg/kg] *(34 patients)* or ketamine [1 mg/kg] *(33 patients)* on the emergence agitation in children operated for ophthalmic surgery under sevoflurane anesthesia. Although, their study showed shorter duration of surgery, the time to extubation was significantly longer in their both groups, compared to the present study. This could be explained by the difference in wash out profile between sevoflurane and desflurane and the relatively high dose of their premedication; midazolam or ketamine with their remarkable hypnotic or dissociative effects, which also may explain the significant reduction in then emotional status following the premedication and the insignificant difference in the overall emergence agitation score between their groups. The midazolam group, in their study, showed significant higher agitation at 10 and 20 min in PACU which was reflected by the higher frequency of midazolam *(61% of patients*) and fentanyl *(88% of patients*) use to control the patients compared to the ketamine group. This finding may be explained by the lack of pre-emptive or preventive analgesia in their study, apart from intraoperative fentanyl (1 μg/kg) at induction, which was compensated by the analgesic effect of ketamine which midazolam lacks. In the presented study, the pre-emptive and preventive subconjunctival bupivacaine and diclofenac suppository may explain the lower frequency of rescue meperidine in the PACU.

Yi Hwa Choi et al., [21] examined the effect of remifentanil *(34 patients)* and remifentanil-alfentanil *(35 patients)* administration on emergence agitation after ophthalmic surgery in children. Their results showed that adding alfentanil prolonged the time to extubation [11.2 ± 2.3 min] compared to remifentanil alone [9.5 ± 2.4 min] or placebo [9.2 ± 2.3 min]. Their prolonged time to extubation compared to the present study could be explained by the respiratory depressant effect of opioids they used, particularly, the use of alfentanil just 10 min before to the end surgery. Again, they depended on neuromuscular blockade which mandates a certain degree f spontaneous neuromuscular recovery before reversing and we used BIS to titrate the inhalational anesthetic, as well. Also, their results showed a higher incidence of EA in the placebo group [64%] compared to remifentanil [32%] and remifentanil-alfentanil [31%]. Although, their results are matched with present study, but the total absence of analgesics in the placebo group raised ethical questions about their methods.

### Limitations of the present study

Our limited experience and few published literature about the use of gabapentin in children to control EA made a difficulty to choose a proper effective and safe dose. Also, the limited flow of patient to our small recently opened center made it difficult to design our study on a larger group of children. We think it may be applicable to repeat a similar study using higher doses of gabapentin and larger groups of patients.

According to the doses used by Edwards L et al., [14] and Gretchen L et al., [8] we recommend that any further similar studies to use a higher dose of gabapentin (10 mg/kg) to examine the possibility of better efficacy with the same safety.

## Conclusion

Oral gabapentin 5 mg/kg reduced the incidence and severity scoring of emergence agitation (by 20%) with more tendencies for sleeping with preserved response to stimuli in PACU.

## References

[1] Kain ZN, Caldwell-Andrews AA, Maranets I (2004). Preoperative anxiety and emergence delirium and postoperative maladaptive behaviors. Anesth Analg.

[2] Vlajkovic GP, Sindjelic RP (2007). Emergence delirium in children: many questions, few answers. Anesth Analg.

[3] Voepel-Lewis T, Malviya S, Tait AR (2003). A prospective cohort study of emergence agitation in the pediatric postanesthesia care unit. Anesth Analg.

[4] Kim J, Kim SY, Lee JH, Kang YR, Koo BN (2014). Low-dose dexmedetomidine reduces emergence agitation after desflurane anaesthesia in children undergoing strabismus surgery. Yonsei Med J.

[5] Lerman J, Hammer GB, Verghese S (2010). Airway responses to desflurane during maintenance of anesthesia and recovery in children with laryngeal mask airways. Paediatr Anaesth.

[6] Sale SM, Read JA, Stoddart PA (2006). Prospective comparison of sevoflurane and desflurane in formerly premature infants undergoing inguinal herniotomy. Br J Anaesth.

[7] Behm MO, Kearns GL (2001). Treatment of pain with gabapentin in a neonate. Pediatrics.

[8] Sacha GL, Foreman MG, Kyllonen K, Rodriguez RJ (2017). The use of gabapentin for pain and agitation in neonates and infants in a neonatal ICU. J Pediatr Pharmacol Ther.

[9] Kong VK, Irwin MG (2007). Gabapentin: A multimodal perioperative drug?. Br J Anesth.

[10] Mizrak A, Erbagci I, Arici T, Ozcan I, Ganidagli S, Tatar G (2010). Ketamine versus propofol for strabismus surgery in children. Clin Ophthalmol.

[11] Mizrak A, Erbagci I, Arici T, Avci N, Ganidagli S, Oner U (2011). Dexmedetomidine use during strabismus surgery in agitated children. Med Princ Pract.

[12] Jung HJ, Kim JB, Im KS, Oh SH, Lee JM (2010). Effect of ketamine versus thiopental sodium anesthetic induction and a small dose of fentanyl on emergence agitation after sevoflurane anesthesia in children undergoing brief ophthalmic surgery. Korean J Anesthesiol.

[13] Aouad MT, Yazbeck-Karam VG, Nasr VG, El-Khatib MF, Kanazi GE, Bleik JH (2007). A single dose of propofol at the end of surgery for the prevention of emergence agitation in children undergoing strabismus surgery during sevoflurane anesthesia. Anesthesiology.

[14] Edwards L, DeMeo S, Hornik CD, Cotten CM, Smith PB, Pizoli C, Hauer JM, Bidegain M (2016). Gabapentin use in the neonatal intensive care unit. J Pediatr.

[15] Pandit UA, Collier PJ, Malviya S, Voepel-Lewis T, Wagner D, Siewert MJ (2001). Oral transmucosal midazolam premedication for preschool children. Can J Anaesth.

[16] Thomas WF, Macario A, Miller RD (2005). The postanesthesia care unit. Anesthesia.

[17] Kaki AM, Al Marakbi W (2008). Post-herniorrhaphy infiltration of tramadol versus bupivacaine for postoperative pain relief: a randomized study. Ann Saudi Med.

[18] Bedirli N, Akçabay M, Emik U (2017). Tramadol vs dexmedetomidine for emergence agitation control in pediatric patients undergoing adenotonsillectomy with sevoflurane anesthesia: prospective randomized controlled clinical study. BMC Anesthesiol.

[19] Amin SM, Amr YM (2011). Comparison between preemptive gabapentin and paracetamol for pain control after adenotonsillectomy in children. Anesth Essays Res.

[20] Kim KM, Lee KH, Kim YH, Ko MJ, Jung JW, Kang E (2016). Comparison of effects of intravenous midazolam and ketamine on emergence agitation in children: randomized controlled trial. J Int Med Res.

[21] Choi YH, Kim KM, Lee SK, Kim YS, Kim SJ, Hwang WS, Chung JH (2016). Effects of remifentanil and remifentanilalfentanil administration on emergence agitation after brief ophthalmic surgery in children. BMC Anesthesiol.

